# High-Fidelity Perforator Visualization for Cadaver Dissection in Surgical Training

**DOI:** 10.1055/s-0043-1771272

**Published:** 2023-08-31

**Authors:** Allen Wei Jiat Wong, Yee Onn Kok, Khong Yik Chew, Bien Keem Tan

**Affiliations:** 1 Plastic Reconstructive and Aesthetic Surgery Service, Sengkang General Hospital, Singapore, Singapore; 2 Department of Plastic Reconstructive and Aesthetic Surgery, Singapore General Hospital, Singapore, Singapore

**Keywords:** cadaver, high fidelity, dissection, perforator

## Abstract

In the first half of the third century B.C., Herophilus and Erasistratus performed the first systematic dissection of the human body. For subsequent centuries, these cadaveric dissections were key to the advancement of anatomical knowledge and surgical techniques. To this day, despite various instructional methods, cadaver dissection remained the best way for surgical training. To improve the quality of education and research through cadaveric dissection, our institution has developed a unique method of perforator-preserving cadaver injection, allowing us to achieve high-fidelity perforator visualization for dissection studies, at low cost and high efficacy. Ten full body cadavers were sectioned through the base of neck, bilateral shoulder, and hip joints. The key was to dissect multiple perfusing arteries and draining veins for each section, to increase “capture” of vascular territories. The vessels were carefully flushed, insufflated, and then filled with latex dye. Our injection dye comprised of liquid latex, formalin, and acrylic paint in the ratio of 1:2:1. Different endpoints were used to assess adequacy of injection, such as reconstitution of eyeball volume, skin turgor, visible dye in subcutaneous veins, and seepage of dye through stab incisions in digital pulps. Dissections demonstrated the effectiveness of the dye, outlining even the small osseous perforators of the medial femoral condyle flap and subconjunctival plexuses. Our technique emphasized atraumatic preparation, recreation of luminal space through insufflation, and finally careful injection of latex dye with adequate curing. This has allowed high-fidelity perforator visualization for dissection studies.

## Introduction

In the first half of the third century B.C., Herophilus and Erasistratus performed the first systematic dissection of the human body. 1 For subsequent centuries, these cadaveric dissections were key to the advancement of anatomical knowledge and surgical techniques. Meticulous lead oxide injection into cadavers enabled Ian Taylor to identify the now-classic angiosomes. 2 3 To this day, despite various instructional methods, cadaver dissection remained the best way for surgical training. 4 5 6 7 The quality of the injected cadavers would have a direct impact on the quality of dissection. Anatomical information could be obscured by leaked dyes, or simply lost to decomposition due to inadequate preservation. To improve the quality of education and research through cadaveric dissection, our institution has developed a unique method of perforator-preserving cadaver injection, allowing us to achieve high-fidelity perforator visualization for dissection studies, at low cost and high efficacy. This technique has allowed us to perform detailed research on anatomy as well as advanced training for our plastic surgery residents and fellows.

## Ideas

Ten fresh full body cadavers were obtained from Science Care (Phoenix, USA) for the purpose of education and research. These bodies were thawed out to room temperature, so that the intraluminal spaces would not be obstructed by ice formation.

### Preparation of Cadavers via Subunits

For better results, we sectioned the cadaver bodies into subunits, at the neck, shoulder, and hip joint. The resulting subunits of the head, trunk, and upper and lower limbs were prepared and injected separately, so that each subunit could be processed separately, to preserve in greater detail traits that were unique to each part.

### Identification and Cannulation of Main Pedicle

The main perfusing arteries and draining veins for each section were dissected and identified. For the head and neck region, the main vessels were the carotid arteries, internal jugular veins, external jugular veins, vertebral arteries, and vertebral veins. For the upper limbs, the main vessels were brachial artery, radial artery, venae comitantes, axillary vein, and distal cephalic vein. For the torso, the main vessels were the subclavian artery, subclavian veins, external iliac artery, and external iliac vein. For the lower limbs, the main vessels were the superficial femoral artery, superficial femoral vein, great saphenous vein, posterior tibial artery, and its venae comitantes. If greater details were needed, we would need to separately process the superficial and deep venous system of each subunit. After identification and exposure of the pedicles, they would be cannulated with 18G intravenous cannulas in the anterograde and retrograde fashion. This would facilitate the next step of intraluminal preparation.

### Intraluminal Preparation

**Fig. 1:**
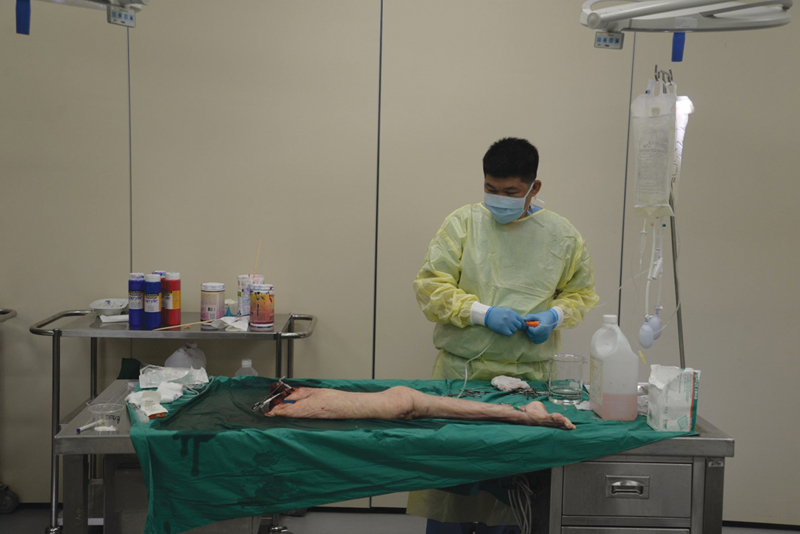
Our laboratory setup for cadaver injection, with drip stands for infusion of the specimen.

After demise, the intraluminal contents of the cadaver would coagulate and form a physical barrier to injection. At the same time, the biological debris would act as a sponge sump, which would adsorb the dye and prevent a homogenous spread of dye. Therefore, it is critical to evacuate the intraluminal space for the best dye results. Saline infusion bags would be connected to the arteries and veins via the 18G intravenous cannula and elevated at 150 cm above the specimen ( Fig. 1 ). This would create a flushing pressure of 150 cm H _2_ O, which would be equivalent to 110 mm Hg. This amount of pressure was chosen as it is physiological and would minimize trauma to the vessels, especially that of small perforators. Depending on the size of the body subunits, around 200 to 400 mL of saline would be used each. If performed correctly, an outflow of debris would be seen at the arteries and veins. If there were resistance or obstruction within the vessel, the saline flush would be performed for the arteries and veins in a retrograde fashion, to dislodge any remaining thrombus by flushing at it from the opposite direction.

Next, using 20-mL Luer-lock syringes connected to the cannula, the arteries and veins were gently insufflated with air to recreate the intraluminal space for the dye. The relatively small size of the syringe would prevent overzealous injection that can cause high pressure. This insufflation of air served to splint vessels open so that there would be less resistance for the next step which would be the injection of latex dye. If done appropriately, one would see bubbling at the opposite end of the vessels. For example, after insufflating through the superficial femoral artery, one will see bubbling from the cannula inserted into the anterior tibia artery.

### Latex Dye Injection

**Fig. 2:**
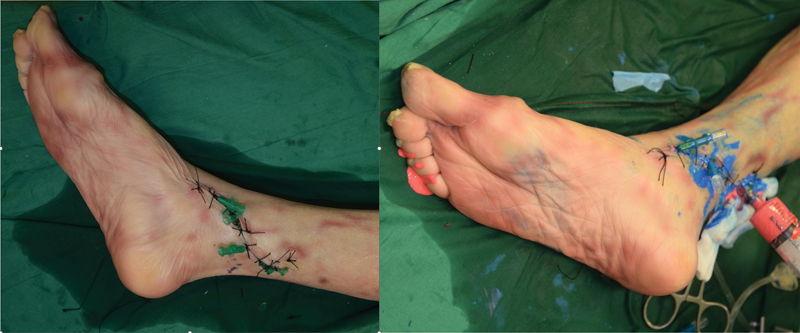
Preparation of the right lower limb cadaveric specimen, showing the various injection cannulas placed into both the posterior tibial artery and veins. The picture on the right showed the endpoint of injection, where the dye could be seen from stab incisions in the toe pulps.

The injection dye formula comprised of a mixture of liquid latex (Castin' Craft Mold Builder Fields Landing, CA), 10% formalin, and acrylic paint (Daler-Rowney, United Kingdom), in the ratio of 1:2:1, respectively. These latex dyes were injected by hand through 20-mL syringes through the intravenous cannula of each vessel. We would use red dye for arteries and blue dye for veins. One could also inject different shades of colors between the superficial and deep venous system to demonstrate the unique territory that each perfused. For veins, injection was done in the anterograde fashion via the intravenous cannula, so that valves would not obstruct the flow of the viscous dye. For example, the dye would be injected vial the great saphenous vein or anterior tibial venae comitantes, from the distal end of the lower limb. Generally, the valves in the cadavers would still be impede retrograde flow, hence retrograde injection may result in excessive pressures that can damage the veins. The volume of latex dye required for each subunit would be approximately 100 to 150 mL of latex dye per arterial or venous vessel. Specific endpoints were observed to determine adequacy of preparation, such as the reconstitution of eyeball volume, restoration of skin turgor, and seepage of dye through skin pricks in the digital pulps ( Fig. 2 ). In thin cadavers, the dye could even be seen to perfuse through superficial veins under the skin.

### Dye Fixation

Deep freezing would be required for the latex dye to solidify and set, allowing easier handling of the tissue during dissection. If the latex dye was not fixed, it would tend to seep around the tissue during the dissection, obscuring fine anatomical details. To fix the latex dye, the cadavers were frozen at below 20 to 40°C for approximately 2 to 5 days. At this temperature range, musculoskeletal tissue could also be preserved for up to 6 months with minimal degradation. 8 Prior to leaving it in the freezer, tourniquets were tied at the open-ending stumps of the cadaveric subunit, to prevent seepage of the dye out of the cadaver. The intravenous cannula would be left in situ and capped to prevent leakage of the dye. It would not be advisable to remove the cannula as it would leave behind a puncture mark within the vessel. This puncture mark would not seal in a cadaver because it would not behave like living tissue, hence causing the dye to leak out from the vessels during fixation. By sealing the stumps and vessels, it would force the dye to stay within the lumen of the vessels, allowing maximal fill of the vessel for visualization with high fidelity. To utilize the cadavers after dye fixation, they would need to be removed from deep freeze (below 20°C), then thawed at 4°C for 48 hours, followed by another 12 hours at room temperature.

### Results

**Fig. 3:**
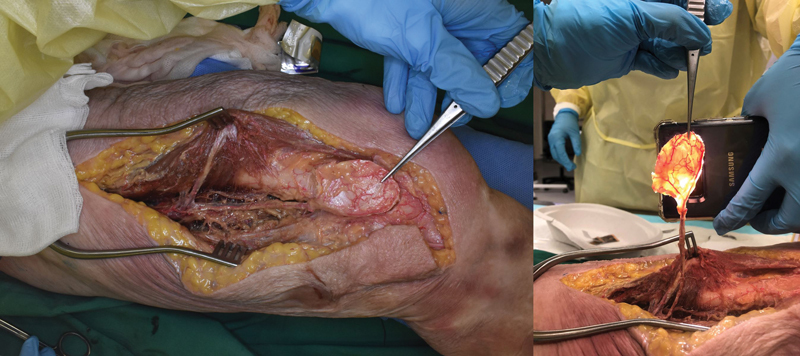
Demonstration of the medial femoral condyle periosteal flap, showing good fill of the periosteal vessels that supply this flap.

**Fig. 4:**
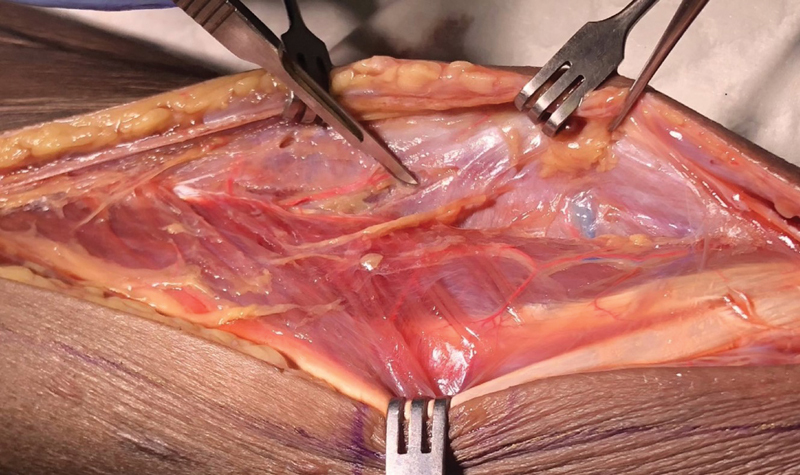
Demonstration of the anterolateral thigh flap, with good fill of small perforators and low leak rate, keeping the dissection field clean and neat.

A total of 10 full body cadavers were prepared and partitioned into subunits for preparation. The bodies were thawed at room temperature for 12 hours prior to injection. An average of 60 minutes was required for full preparation for one upper limb, and an average of 90 minutes was required for full preparation of one lower limb. After adequate period of dye fixation, the cadavers were dissected to evaluate the technique. The vascular trunk was well filled with latex dyes, from large named vessels to intramuscular branches and skin perforators. Even the small periosteal vessels of the medial femoral condyle periosteal flap could be seen ( Fig. 3 ). The latex also did not seep out or become too runny, maintaining the cleanliness of the dissection field ( Fig. 4 ).

## Discussion

Cadaver dissection remained as one of the best methods for surgical training. 5 Cadaver dissection would provide the most anatomically correct experience, including accurate tactile feedback to the learner. Such lessons could not be imparted by silicon or artificial models. Also, anatomical variances were random and unpredictable events. Only cadavers could recreate such anatomical variants that would be useful for training and research. Thus, short of vivisecting live patients, cadaver dissection would continue to be the best method for surgical training and research. Therefore, we required an easily reproducible method of dye injection that would best preserve the vessel quality, and visibility, down to the perforator level, allowing a high-fidelity visualization of anatomy. We would elaborate on key principles behind our cadaver injection technique below.

### Atraumatic Preparation of Cadavers

First, the atraumatic preparation of the cadavers was key to the preservation of anatomical details. The first step was to partition the body into smaller manageable units. This avoided injection of the entire body at one go, which would have resulted in injection of large volumes at higher pressure. This would result in inadvertent damage to anatomical structures, especially that of finer caliber perforators.

After death, organic debris would accumulate within the vessel lumens, preventing the latex dye from filing up the same space. A low-pressure saline flush would be required to clear these debris without damaging the vessels. If the vessels were damaged, the eventual latex dye would seep out and contaminate the surrounding tissue, making dissection a messy affair. Using saline bags elevated to 150 cm above the cadaver would produce a physiological pressure of approximately 110 mm Hg, reducing potential damage to the vessels. This simple application of physics would also obviate the need for injection pumps that are costly or avoid the use of special magnetic devices to push fluid along. 9 10 In the case of veins, the valvular patency has to be considered. Retrograde preparation and infusion of the vein may not be possible and could even rupture the vein due to the presence of valves. Hence, we advise anterograde preparation for veins, in the direction of distal limb toward proximal limb.

### Recreating Luminal Space with Insufflation

After flushing the debris from the vessels, the lumen would tend to collapse due to low internal pressure. This would create high resistance in the vessel, which would require higher injection force to push the latex dye through. Doomernik et al even had to use an injection pressure that was equivalent to 2,000 cm H _2_ O to inject their cadavers. 10 Such high pressure would certainly damage some vessels inadvertently. To minimize this damage, the air would need to be gently and smoothly injected via a 20-mL syringe, making sure to stop whenever there was excessive resistance. In vessels with excessive resistance, one would need to repeat the saline flushes to clear the intraluminal obstructions. The novel concept of air insufflation was not described before, but was key to allowing smooth, even latex dye injection.

### Latex Dye and Fixation

Many different types of injection fluid had been attempted for cadaver preparation, including acrylic paint, latex, gelatin, silicon, Araldite F, and Batson's no. 17. 9 10 Acrylic paint, by itself, did not cure well, and tended to leak into surrounding tissue very easily. Gelatin was water-soluble but lost its color intensity fast. Liquid silicone was expensive but did not have good color intensity. Araldite F was too viscous in its liquid form, making injection difficult. Adding hardener to Araldite F also caused an exothermic reaction that even melted a plastic stirrer and three-way valve. 10 Batson's no. 17 was toxic and required a fumigation hood. Watanabe et al described a method of using magnetic dye 9 to help improve uptake of dye into the cadaver, but we found that such methods were expensive, and would be difficult to obtain. Latex was a cheap, water-soluble, flexible, and elastic product; hence, it was our preferred choice. To preserve the quality of the cadaver, we mixed latex with formaldehyde. To add color to the latex, we added acrylic paint. Our injection dye formula comprised of a mixture of liquid latex, formalin, and acrylic paint in the ratio of 1:2:1. We find this injection dye to be cheap, effective, and relatively nontoxic.

To fix the latex dye, the cadavers are frozen at below 20 to 40°C for approximately 2 to 5 days. This temperature was chosen because it was also the temperature range recommended for storage of musculoskeletal allografts by the American Association of Tissue Banks, and it allowed for preservation without degradation for 6 months. 8 This would then serve the dual purpose of setting the latex dye and preserving the cadavers for high-fidelity visualization.

The advantages of our injection technique lie in high-fidelity visualization of perforators, low cost, and easy replication in other institutions.
